# Metabolic Aging as an Increased Risk for Chronic Obstructive Pulmonary Disease

**DOI:** 10.3390/metabo14120647

**Published:** 2024-11-21

**Authors:** Claire J. Guo, Suneeta Godbole, Wassim W. Labaki, Katherine A. Pratte, Jeffrey L. Curtis, Robert Paine, Eric Hoffman, Meilan Han, Jill Ohar, Christopher Cooper, Katerina J. Kechris, Dawn L. DeMeo, Russell P. Bowler

**Affiliations:** 1 Department of Biostatistics and Informatics, Colorado School of Public Health, University of Colorado Anschutz Medical Campus, Aurora, CO 80045, USA; claire.guo@cuanschutz.edu (C.J.G.); suneeta.godbole@cuanschutz.edu (S.G.); katerina.kechris@cuanschutz.edu (K.J.K.); 2 Division of Pulmonary and Critical Care Medicine, University of Michigan, Ann Arbor, MI 48109, USA; wlabaki@med.umich.edu (W.W.L.); jlcurtis@med.umich.edu (J.L.C.); 3 Division of Biostatistics and Bioinformatics, National Jewish Health, Denver, CO 80206, USA; prattek@njhealth.org (K.A.P.); mrking@med.umich.edu (M.H.); 4 Medical Service, Veterans Affairs Ann Arbor Healthcare System, Ann Arbor, MI 48105, USA; 5 Division of Respiratory, Critical Care and Occupational Medicine, University of Utah, Salt Lake City, UT 84132, USA; robert.paine@hsc.utah.edu; 6 Department of Radiology, University of Iowa, Iowa City, IA 52242, USA; eric-hoffman@uiowa.edu; 7 Section of Pulmonary, Critical Care, Allergy and Immunologic Diseases, Department of Internal Medicine, Wake Forest School of Medicine, Wake Forest University, Winston-Salem, NC 27101, USA; johar@wakehealth.edu; 8 David Geffen School of Medicine, University of California Los Angeles, Los Angeles, CA 90095, USA; ccooper@mednet.ucla.edu; 9 Channing Division of Network Medicine, Brigham and Women’s Hospital, Boston, MA 02115, USA; ddemeo@bwh.harvard.edu; 10 Genomic Medicine Institute, Cleveland Clinic, Cleveland, OH 44106, USA

**Keywords:** aging, COPD, metabolic age

## Abstract

Background/Objectives: Both aging and chronic obstructive pulmonary disease (COPD) are strongly associated with changes in the metabolome; however, it is unknown whether there are common aging/COPD metabolomic signatures and if accelerated aging is associated with COPD. Methods: Plasma from 5704 subjects from the Genetic Epidemiology of COPD study (COPDGene) and 2449 subjects from Subpopulations and intermediate outcome measures in COPD study (SPIROMICS) were profiled using the Metabolon global metabolomics platform (1013 annotated metabolites). Post-bronchodilator spirometry measures of airflow obstruction (forced expiratory volume at one second (FEV_1_)/forced vital capacity (FVC) < 0.7) were used to define COPD. Elastic net regression was trained on never and former smokers with normal spirometry and no emphysema to create a metabolomic age score which was validated in SPIROMICS subjects. Results: Our metabolic age score was strongly associated with chronic age in the validation cohort (correlation coefficient = 0.8). COPD subjects with accelerated aging (>7 years difference between metabolic and actual age) had more severe disease compared with those who had decelerated aging (<−7 years difference between metabolic and actual age). COPD and aging metabolites were shared more than expected (*p* < 0.001), with amino acid and glutathione metabolism among pathways overrepresented. Conclusions: These findings suggest a common mechanism between aging and COPD and that COPD is associated with accelerated metabolic aging.

## 1. Introduction

Persistent respiratory symptoms and airflow limitation due to chronic obstructive pulmonary disease (COPD) makes the disease a strong negative impactor of quality of life as well as a leading cause of death and hospitalizations both worldwide and in the United States [1,2]. These symptoms are usually caused by repeated exposure to noxious particles or gases, including cigarette smoke, that damage the airways and lungs, ultimately resulting in airway and/or alveolar abnormalities [3]. COPD is also characterized by many comorbidities, including muscle wasting, cardiovascular disease, osteoporosis, and depression [4]. This suggests that COPD is a generalized systemic disease not just limited to the lungs and that there are subsequent downstream changes to molecular pathways, including disturbances to the metabolome. Many of these comorbidities are also associated with age; however, COPD patients often present with these comorbidities earlier than those at a similar age without COPD, indicating that COPD might be a disease of early aging [5]. In this study, we examine the metabolomic signatures of COPD and aging to identify the shared and distinct pathologic signatures of each disease. 

The aging metabolome is now well established and includes changes in energy metabolism (e.g., decreasing nicotinamide adenine dinucleotide (NAD+)), lipids (e.g., those carried by low-density lipoprotein (LDL) particles, such as ceramides and sphingomyelins), and amino acids [6]. A 2014 large metabolomic study of individuals of Northern European ancestry observed significant differences in males and females for lipoproteins, cholesterol, and triglyceride levels, whereas atherogenic metabolites and certain amino acids were only found to be increased in females during the time of menopausal transition [7]. A smaller (n = 30) untargeted metabolomic study found that antioxidants and compounds associated with high physical activity (e.g., carnosine, uridine diphosphate (UDP)-acetyl-glucosamine, NAD+, and leucine) declined with age, whereas metabolites that increased with age included those that were associated with a decline in renal and liver functions [8]. In 2016, another, more comprehensive liquid chromatography-tandem mass spectrometry (LC-MS/MS) study of 2578 plasma metabolites showed that there were age-related metabolites that were gender- and non-gender-specific, including multiple lipid species that decreased with aging [9]. These studies illustrate some of the systemic age-related metabolic changes that are common across multiple different metabolomic platforms.

The relationship between individual metabolites and COPD has also been explored, particularly for spirometry tests of lung function. Specifically, three metrics obtained from these tests are typically used: forced expiratory volume in 1 s (FEV_1_), FEV_1_ percentage of predicted (FEV_1_%), and the FEV_1_/Forced Vital Capacity (FVC) ratio (FEV_1_/FVC) (see review [10]). Multivariate models using more than one metabolite have also been used to predict COPD. Pinto-Plata et al. used machine learning methods to identify distinct plasma metabolomic profiles between survivors and non-survivors of COPD two years prior to their death [11]. These profiles were also characterized by differences in energy metabolism pathways. In Godbole et al. 2022 [12], metabolomic scores with 132 and 129 metabolites were used to predict FEV_1_ and emphysema, respectively. These models performed better than models with clinical covariates alone. COPD metabolites were enriched in arginine biosynthesis; aminoacyl-tRNA biosynthesis; and glycine, serine, and threonine metabolism. Others have created metabolomic scores to predict acute COPD exacerbations [13] as well as a number of other diseases, including coronary heart disease [14,15], type 2 diabetes [16], incident heart failure [17], and pulmonary arterial hypertension [18].

Regularized regression models, such as elastic net, have been used by others to create -omic age scores. This began with Horvath [19] and Hannum et al. [20], who both trained elastic net models on transcriptomic data to predict age, arriving at transcriptomic age scores. Horvath found accelerated age or transcriptomic age scores greater than chronological age in cancer tissues. Hannum et al. found that the rate of transcriptomic age was impacted by gender and genetic variants. Since then, many have created age scores using a variety of -omic markers, and the concept has been named “biological” age. Many have also found that accelerated biological age is associated with a number of health outcomes. These studies have been reviewed in Rutledge et al. [21] and Li et al. [22]. Although elastic net has been used to create age and COPD metabolite scores, there have been no investigations into the overlap between the two.

This study investigates the overlap between the aging and COPD metabolomes and addresses some of the limitations—in sample size, diversity, and number of metabolites examined—of previous studies by using two independent, well-phenotyped case–control populations with 8153 subjects whose comprehensive plasma metabolomic profiles included > 1000 annotated metabolites. The study is the largest COPD metabolomic study to date. It also creates a normal lung function aging score as well as a COPD metabolome score and then contrasts the metabolites that make up aging scores with COPD scores to identify common and distinct aging and COPD metabolic features, as well as to identify COPD subjects who have accelerated aging.

## 2. Materials and Methods

### 2.1. Cohorts

The Genetic Epidemiology of COPD (COPDGene) (ClinicalTrials.gov Identifier: NCT00608764) is an NIH-sponsored multicenter cohort of never, former, and current smokers aged 45–80 years old. Written informed consent was obtained from all subjects involved in the study. Details of the COPDGene study are provided elsewhere [23]. In brief, this study enrolled 10,198 non-Hispanic White and African American participants with at least 10 pack-years of smoking and 465 individuals with no smoking history. Metabolomic profiling was performed on 5704 participants from the second visit of the study.

The Subpopulations and Intermediate Outcome Measures in COPD Study (SPIROMICS) (ClinicalTrials.gov Identifier: NCT01969344) is an NIH-sponsored multicenter cohort of never, former, and current smokers aged 40–80 years old. Written informed consent was obtained from all subjects involved in the study. Details of the cohort are provided elsewhere [24]. In brief, this study recruited 2771 participants with at least 20 pack-years of smoking and 202 participants who were never smokers. In total, 73% of the participants self-identified as non-Hispanic White, and 2449 had plasma available for metabolomic profiling of baseline fasting blood samples. Metabolomic profiling as performed on the baseline blood draw of the study.

### 2.2. Clinical Data and Definitions

COPD was defined using post-bronchodilator FEV_1_/FVC, with a case defined as FEV_1_/FVC < 0.7 [25]. Emphysema was quantitated by CT scan at total lung capacity using low attenuation area (LAA) voxels < −950 Hounsfield units [26]. At the time of a study visit, a never smoker was defined as someone who smoked < 100 lifetime cigarettes [27], and a former smoker was defined as someone who had quit smoking and not smoked a cigarette in the past 30 days; these definitions are used to define the categories in the rows of Table 1. For the purposes of developing our metabolomic age score in this analysis, we defined our control subjects as those with normal spirometry (GOLD 0) grade (FEV_1_ ≥ 80% and FEV_1_/FVC ≥ 0.7), no emphysema by LAA < 5%, and never smokers or former smokers who stopped smoking at least 5 years ago; these grouping are show in the columns of Table 1. 

### 2.3. Metabolomic Profiling and Processing

Plasma samples from both cohorts were profiled using the Metabolon global metabolomics platform (Durham, NC, USA), as described previously, although profiling for each cohort occurred approximately 6 months apart [12]. Metabolites are annotated by Metabolon based on level of confidence that the compound measured matches the intended metabolite using an authentic chemical standard; a single asterisk (*) after a metabolite’s name indicates that the compound most likely matches but has not yet been confirmed with a standard, a double asterisk (**) indicates there is reasonable confidence of a match but a standard is not available for confirmation, and no asterisk indicates a confirmed match. Metabolite values were batch-normalized, within each study, by dividing by the median metabolite value for each metabolite within a batch. After batch normalization, metabolite principal components showed a significant reduction in association with batch, so no further normalization was needed [28]. For this analysis, metabolites were excluded if missing in >20% of subjects, and we only examined metabolites that had the same missing pattern in both studies (e.g., mediations and nicotine metabolites). This resulted in 831 metabolites of the 1314 metabolites identified in both studies. The missing values of these 831 metabolites were then imputed with k nearest-neighbor imputation (kNN; k = 10) using the R package ‘impute’ and then log_2_-transformed for all analyses. The other metabolites that were excluded from the analysis had discrepant missing patterns: 122 metabolites had <20% samples missing in one study and 20–80% missing in the other, and 361 metabolites had at least one cohort with ≥80% missing samples.

### 2.4. Software and Statistical and Bioinformatic Analysis

The statistical software R (R Foundation for Statistical Computing, Vienna, Austria) version 4.0.2 was used for all analyses. The R package glmnet was used for ElasticNet, stats was used for overrepresentation analysis and univariate linear models, and table1 was used to create characteristic tables. The coefficients from ElasticNet were used as weights to develop a final score and were interpretable in terms of the linear relationship between the metabolite and the outcome. For the aging score, we wished to create a covariate free score with all subjects who did not have lung disease so that it could be applied without gender or race biases, even if larger studies that stratify for gender, race, and smoking may result in different metabolites being selected and different weights being applied. Thus, the elastic net model for age was trained on COPDGene never and former smokers (subjects who quit at least 5 years ago and had less than 5% emphysema and normal spirometry). We then tested the score on SPIROMICS subjects who met the same criteria. The final score was then applied to other independent former and current smokers in each of the cohorts, looking separately at those without obstruction (GOLD 0) and with less than 5% emphysema (described as current/former smokers without COPD and emphysema) compared to those who did not meet those criteria (described as current/former smokers with COPD or emphysema). For the lung obstruction score, all COPDGene metabolite data were used to train an elastic net model predicting continuous FEV_1_/FVC. It was then tested on all SPIROMICS data. For both scores, 10-fold cross validation was performed on the training data set to obtain the penalty parameter (λ), which corresponds to the largest lambda that minimizes MSE plus one standard error. This was iterated across alpha values of 0 to 1 at a 0.05 interval to identify the alpha that produced the best-performing model. The metabolite score models did not include clinical covariates. Predictions were created with the metabolite coefficients and the set of metabolites selected by the ElasticNet procedure.

To assess the difference between metabolomic age and actual age, a spline was fitted between age_actual_ and age_metabolomic_ using the smooth.spline function from the stats R package. The age difference was then calculated by age_metabolomic_ − age_spline_. To compare approximately 10% of COPD and emphysema subjects who demonstrated the most accelerated or decelerated metabolic age, we used a cutoff of 7 years. Specifically, subjects with accelerated age were defined as age_metabolomic_ − age_spline_ > 7 years and those with decelerated age as age_metabolomic_ − age_spline_ < −7 years. We examined differences in the demographic and clinical characteristics between these two groups, using a *t*-test for continuous variables and a chi-squared test for categorical/binary variables. This was repeated separately in current smokers without COPD and emphysema. A chi-squared test was used to test the probability that the overlap in metabolites happened by chance. Approximately 9% of the total subjects were never smokers with evidence of emphysema or COPD and were not included in the analysis because of their small sample size.

### 2.5. Pathway Analysis

Pathway analysis was conducted by a Fisher’s test to determine overrepresentation of sub- and super-pathways based on metabolites of interest. The sub- and super-pathways used were annotated and provided by Metabolon (Durham, NC, USA). In total, 129 of the 831 metabolites used in our analysis have not been annotated yet. The Fisher’s test *p*-values were also adjusted using the Benjamini–Hochberg procedure to account for multiple testing [29].

### 2.6. Univariate Associations

To identify metabolites associated with age and FEV_1_/FVC, simple univariate linear models without any other covariates were conducted with each phenotype. For association with FEV_1_/FVC, all subjects were used for analysis. The analysis with age was repeated separately in never/former smokers without emphysema or COPD and in current/former smokers with COPD or emphysema.

## 3. Results

### 3.1. Demographic Characteristics

Demographics and clinical characteristics for the COPDGene never/former smokers without COPD or emphysema training, SPIROMICS never/former smokers without COPD or emphysema testing, and other COPDGene and SPIROMICS former- and current-smoker application groups are presented in Table 1. In general, the current smokers without COPD or emphysema were slightly younger, more likely to be women and African American, and less likely to have coronary artery disease (*p* < 0.001).

### 3.2. Metabolomic Age Score

**Figure 1:**
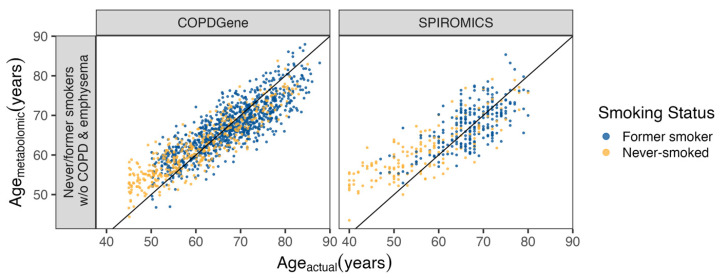
Metabolite age score. The age model was trained on COPDGene never (yellow) and former (blue) smokers without COPD and emphysema (**left** panel) and tested on SPIROMICS never and former smokers without COPD and emphysema (**right** panel). The subjects’ actual ages are on the *x*-axis and predicted metabolomic ages are on the *y*-axis. Former smokers were defined as having stopped smoking at least 5 years ago. Elastic net was used to define the metabolomic age score.

Elastic net (alpha 0.05) selected 378 metabolites for the age model. When testing this model using independent never- and former-smoker control subjects without COPD or emphysema, the predicted–actual age correlations were 0.875 in COPDGene and 0.806 in SPIROMICS (Figure 1). Leucine, Isoleucine, and Valine Metabolism was the most overrepresented sub-pathway, and Amino Acids was the most overrepresented super-pathway (Supplementary Table S1B,C).

### 3.3. Differences Between COPD Subjects with Accelerated and Decelerated Metabolomic Age

**Figure 2:**
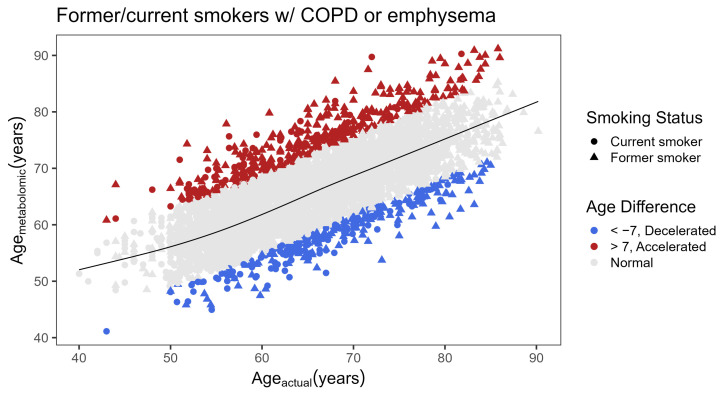
Age acceleration in former (triangle) and current (circle) smokers with COPD or emphysema. A spline was fitted between age_actual_ and age_metabolomic_ (black line). Age difference was calculated by age_metabolomic_ − age_spline_, where subjects having accelerated metabolomic age (red) were those with a difference greater than 7 years and those having decelerated age (blue) were those with a difference less than −7 years. Former smokers were defined as having reported stopping smoking in the past 30 days.

To evaluate whether metabolic age difference was associated with COPD, a spline was fit between actual and metabolomic age (Figure 2). Current and former smokers with COPD or emphysema with accelerated age (age_metabolomic_ − age_spline_ > 7) were compared to those with decelerated age (age_metabolomic_ − age_spline_ < −7) (Table 2). Using a spline allowed us to compare subjects on each tail end of the age difference within the same age group more equivalently, particularly younger subjects for whom the sample size was smaller. Age_spline_ did not differ greatly from Age_actual_, with a mean difference of 0.5462 years and 25th and 75th percentiles of −1.6768 and 2.6501 years, respectively. Those with accelerated metabolic age were more likely to be White (*p* < 0.001), female (*p* < 0.001), former smokers (*p* < 0.001), have more severe COPD by GOLD grade (*p* < 0.001), worse spirometry and more emphysema (*p* < 0.001), and to have higher rates of diabetes and cardiological comorbidities. The same comparison in current smokers without COPD or emphysema (Supplementary Table S2 and Supplementary Figure S1) showed that subjects without COPD or emphysema with accelerated metabolic age were also more likely to be White and female. They also were more likely to have had a stroke and chronic bronchitis, as well as greater rates of other comorbidities that did not reach statistical significance.

### 3.4. A Metabolomic Lung Obstruction Score

**Figure 3:**
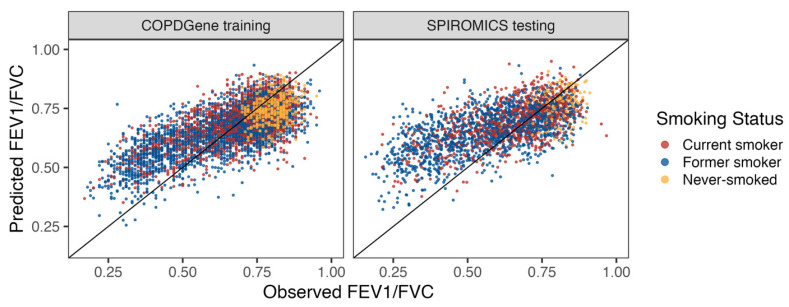
Metabolite-based lung obstruction score. Shown on the *x*- and *y*-axis are observed and predicted FEV_1_/FVC values, respectively. COPDGene (**left**) was used as the training dataset (cor = 0.705) and SPIROMICS (**right**) as the testing dataset (cor = 0.651), with an alpha of 0.1. Former smokers were defined as having reported stopping smoking in the past 30 days.

We next evaluated metabolites associated with obstruction on spirometry. A total of 461 metabolites were selected by elastic net, with an alpha of 0.1 for the lung obstruction metabolomic score model. Correlations between actual and metabolome-predicted FEV_1_/FVC were 0.705 in COPDGene training and 0.651 in SPIROMICS testing (Figure 3).

### 3.5. Overlap Between the Age and COPD Metabolome Scores

**Figure 4:**
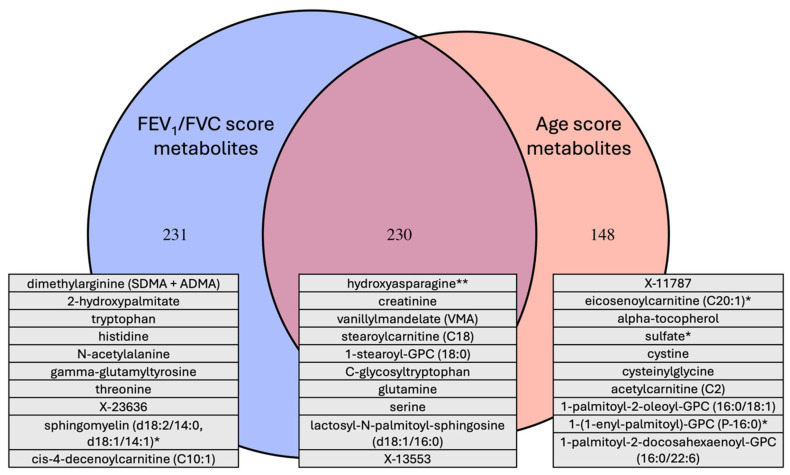
Overlap between metabolites in the COPD and age metabolite score models. There were 230/831 common to both models, 231/831 included only in the COPD model, and 148/831 included only in the age model. The 10 largest beta-coefficient metabolites for age (overlap and age only) or obstruction (FEV_1_/FVC score) are shown. A single asterisk (*) after a metabolite’s name indicates that the compound most likely matches but has not yet been confirmed, a double asterisk (**) indicates there is reasonable confidence of a match, and no asterisk indicates a confirmed match.

Between the elastic net models of age and lung obstruction, there was a higher-than-expected-by-chance overlap in the metabolites selected (Figure 4; *p* < 0.001). Amino acids were overrepresented in both the lung obstruction and age models, although different sets of amino acids were identified in each case (Supplementary Table S1C,E). For example, metabolites in the FEV_1_/FVC model but not in the aging model included dimethylarginine and specific sphingolipids and ceramides. Metabolites in both models included creatinine, urea, and cortisol.

### 3.6. Overlap Between Metabolite Univariate Associations with Age and COPD

**Figure 5:**
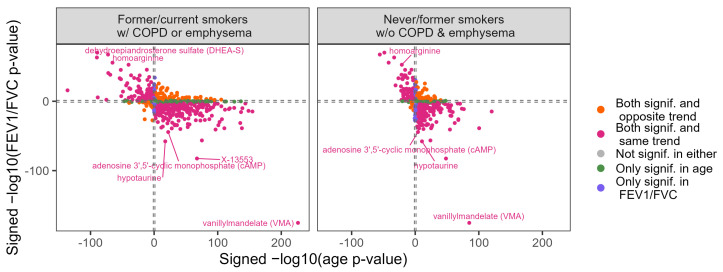
Overlap of metabolite associations with age and lung obstruction in never and former smokers without COPD or emphysema (**left** panel) and in former and current smokers with COPD or emphysema (**right** panel). The sign of the coefficient estimate was multiplied by the −log_10_(*p*-value), with the *x*-axis showing results for univariate metabolite association with age and the *y*-axis showing results for univariate metabolite association with FEV_1_/FVC. Dashed lines at log_10_(0.05) and −log_10_(0.05) on both axes distinguish proteins that were statistically significant.

In general, most metabolites univariately associated with age were also associated with lung obstruction, with similar trends and *p*-values (Figure 5, Supplementary Table S1F). Examples of the metabolites that were higher with age and obstruction are vanillylmandelate, hydroxyasparagine, and arabonate/xylonate. Others, such as androstenodiol and homoarginine, were lower with age and amount of obstruction. Another striking metabolite that was associated with both COPD and age was adenosine 3′, 5′ cyclic monophosphate (cAMP). The metabolites that were significant and concordant for both age and COPD were highly enriched in the Androgenic Steroids sub-pathway and in the Nucleotide super-pathway (Supplementary Table S1G,H). Only a few significant metabolites were significant only for age or COPD or were discordant with age and obstruction severity. Those significant only for age include 3-methylglutaconate, N,N-dimethyl-pro-pro, dimethylarginine, and urea. Those only significantly associated with FEV_1_/FVC include valine and methyl-4-hydroxybenzoate sulfate. The metabolites 2-O-methylascorbic acid, myo-inositol, choline, and tetrahydrocortisol glucuronide were among those that were significantly associated with both age and FEV_1_/FVC, but with trends in discordant directions. These discordant metabolites were enriched in several sub-pathways, including the Urea Cycle, Arginine and Proline Metabolism (*p*-value = 0.002), and Corticosteroids (*p*-value = 0.014), although these were no longer significant following multiple-testing corrections (Supplemental Table S1I).

## 4. Discussion

COPD is a disease that occurs primarily in older persons. While COPD has been postulated to be a disease of accelerate lung aging for some time [30], this is the first study to show that many of the metabolic signatures of COPD and age overlap and that accelerated aging is associated with worse COPD. Although aging is a complex multisystem process, López-Otín et al. described aging in reference to multiple hallmarks of aging. These hallmarks include genomic instability, telomere attrition, epigenetic alterations, loss of proteostasis, deregulated nutrient sensing, mitochondrial dysfunction, cellular senescence, stem cell exhaustion, and altered intercellular communication [31,32]. Some of these features have been described in COPD. For instance, other biomarkers of aging, such as telomere shortening [33] and oxidative damage to DNA [34,35] and the mTOR pathway [36,37], are associated with both aging and COPD or emphysema. In this study, we also found evidence for shared pathology with deregulated nutrient signaling, with many amino acids and carnitines being associated with both age and COPD. We also found evidence for altered intercellular signaling, as with cAMP, androstenodiol, and sphingolipids. cAMP can suppress key inflammatory responses and is the target of phosphodiesterase (PDE) inhibitors, which represent one of the most significant signaling pathways in COPD and are the target of FDA-approved mediations [38]. The strongest positive association between age and COPD concerned VMA, which is a metabolite of catecholamines and may reflect elevated physiologic or psychologic stress [39].

Dimethylarginine (DMA) was one of 231 metabolites selected in the lung obstruction score but not in the age score. DMA is a product of methylated arginine residue protein degradation and may play a role in inflammation through inhibition of nitric oxide synthases [40]. We have previously reported an association with DMA and COPD (in Godbole et al. 2022) as well as the arginine pathway. As we noted in Godbole et al. 2022, several small studies and mouse models have also identified this relationship [40,41,42,43,44]. Other strong markers for both age and COPD were anabolic steroids such as androstenodiol, albeit in an opposite direction, where lower levels were associated with more severe COPD and older age. Lower levels of these hormones may explain why both older age and COPD are associated with lower muscle mass.

We identified several metabolites that had significant univariate associations only with COPD or were significantly associated with both age and COPD but in discordant directions. These findings point to some biological processes unique to COPD separate from healthy aging. Those only significantly associated with FEV_1_/FVC include valine and methyl-4-hydroxybenzoate sulfate. Valine, involved in BCAA degradation, has previously been found to distinguish between patients with advanced COPD (GOLD grade 4) and healthy controls and is associated with increased systemic inflammation [45]. The discordant metabolites included 2-O-methylascorbic acid, myo-inositol, choline, and tetrahydrocortisol glucuronide and represented the Urea Cycle, Arginine and Proline Metabolism, and Corticosteroid sub-pathways.

While our results point to multiple pathways, there are some potential implications regarding how this work might translate into better care and treatment for COPD patients. In general, our biomarkers might suggest that COPD patients and aging-related diseases might improve with lifestyle modifications, such as reducing stress (VMA), improving nutrition (amino acids and carnitines), minimizing inflammation (cAMP, sphingolipid, and arginine pathways), and improving the aging endocrine system (anabolic steroids). These lifestyle modifications may be more beneficial compared to targeted anti-aging molecules, such as resveratrol and pterostilbene, and flavonoids, such as quercetin and fisetin, as well as oleic acid, which have not yet been shown to prevent aging in well-designed randomized clinical trials.

A major limitation of this study is that it is observational, and thus is not clear whether any of these pathways can be modified to prevent COPD progression. For instance, there have been mixed results with androgenic steroids in treating COPD, and mTOR-targeted interventions have not been successful for COPD. Additionally, diminished energy metabolism, as evidenced by amino acids and carnitine pathways, may be secondary to the general inactivity of COPD patients, possibly due to ventilatory limitation, which prevents normal energy use. It would be useful to see if these biomarkers improve after COPD patients undergo combined physical and nutritional rehabilitation. We did not use any clinical or demographic covariates in our analyses, which could potentially have changed the performance of the age and obstruction scores and changed the results of the univariate associations. However, our univariate associations can provide a sense of metabolomic correlations with lung obstruction and age without the effect of covariates. We also did not adjust for lifestyle factors, including diet, exercise, or medications, which also could have strong effects on the metabolome. Both our age and lung obstruction scores had poorer performance at the lower range of each outcome. This is likely due to smaller sample sizes for younger subjects and subjects with strong lung obstruction, and the results would likely improve with greater sample sizes.

## 5. Conclusions

This study provides strong molecular evidence for shared pathophysiology of aging in COPD, including diminished nutritional functioning (amino acids and carnitine), impaired androgenic signaling, and increased stress and inflammation (VMA, sphingolipids, and DMA). People who have accelerated metabolic aging also have more severe COPD; thus, any interventions that reduce aging might also diminish COPD progression.

## Figures and Tables

**Table 1:** Characteristics of COPDGene and SPIROMICS subjects.

	Never/Former Smokers Without COPD or Emphysema		Current Smokers Without COPD or Emphysema		Former/Current Smokers with COPD or Emphysema		*p*-Value
	COPDGene (N = 1346)	SPIROMICS (N = 413)	COPDGene (N = 818)	SPIROMICS (N = 323)	COPDGene (N = 3540)	SPIROMICS (N = 1681)	
Age (years), mean (SD)	65.4 (9.25)	62.9 (9.51)	59.1 (6.33)	55.2 (8.82)	66.2 (8.56)	65.0 (8.05)	<0.001
Race White/Black/other, %	87.4/12.6/0	81.8/12.3/5.9	42.7/57.3/0	55.7/38.7/5.6	71.3/28.7/0	80.5/15.2/4.3	<0.001
Gender, male, n (%)	582 (43.2%)	181 (43.8%)	387 (47.3%)	154 (47.7%)	1888 (53.3%)	945 (56.2%)	<0.001
Smoking Status Never/former/current, %	29.3/70.7/0	40/60/0	0/0/100	0/0/100	0/65.2/34.8	0/67.3/32.7	NA
Num. recent exacerbations	0 (0, 1.00)	0 (0, 1.00)	0 (0, 1.00)	0 (0, 2.00)	0 (0, 2.00)	0 (0, 2.00)	<0.001
GOLD stage, n (%)							NA
GOLD 0	951 (70.7%)	248 (60.0%)	818 (100%)	323 (100%)	514 (14.5%)	130 (7.7%)	
GOLD 1	0 (0%)	0 (0%)	0 (0%)	0 (0%)	507 (14.3%)	334 (19.9%)	
GOLD 2	0 (0%)	0 (0%)	0 (0%)	0 (0%)	1063 (30.0%)	667 (39.7%)	
GOLD 3	0 (0%)	0 (0%)	0 (0%)	0 (0%)	565 (16.0%)	347 (20.6%)	
GOLD 4	0 (0%)	0 (0%)	0 (0%)	0 (0%)	235 (6.6%)	142 (8.4%)	
PRISm	12 (0.9%)	0 (0%)	0 (0%)	0 (0%)	656 (18.5%)	61 (3.6%)	
Never smoker	383 (28.5%)	165 (40.0%)	0 (0%)	0 (0%)	0 (0%)	0 (0%)	
FEV_1_ (liters)	2.71 (0.700)	2.85 (0.697)	2.69 (0.671)	2.89 (0.704)	1.86 (0.792)	1.86 (0.823)	NA
Emphysema, %	1.59 (1.85)	1.64 (1.39)	0.883 (1.06)	0.997 (0.903)	8.01 (10.6)	10.4 (11.2)	NA
FVC (liters)	3.45 (0.876)	3.62 (0.882)	3.45 (0.884)	3.71 (0.908)	2.98 (0.967)	3.38 (1.06)	NA
FEV_1_/FVC	0.787 (0.0493)	0.788 (0.0505)	0.784 (0.0487)	0.782 (0.0487)	0.616 (0.151)	0.542 (0.147)	NA
History of diabetes, n (%)	169 (12.6%)	54 (13.1%)	125 (15.3%)	25 (7.7%)	670 (18.9%)	231 (13.7%)	<0.001
History of stroke, n (%)	20 (1.5%)	14 (3.4%)	28 (3.4%)	10 (3.1%)	130 (3.7%)	66 (3.9%)	0.00323
History of heart attack, n (%)	56 (4.2%)	17 (4.1%)	32 (3.9%)	5 (1.5%)	244 (6.9%)	123 (7.3%)	<0.001
History of coronary artery disease, n (%)	86 (6.4%)	24 (5.8%)	32 (3.9%)	7 (2.2%)	346 (9.8%)	172 (10.2%)	<0.001
Chronic bronchitis, n (%)	46 (3.4%)	30 (7.3%)	116 (14.2%)	74 (22.9%)	643 (18.2%)	367 (21.8%)	<0.001

Exacerbations included those treated with antibiotics and/or corticosteroids in the 12 months prior to the visit; shown are n (percentages), means (standard deviations), or medians (5th, 95th percentiles); spirometry volumes are in post-bronchodilator therapy and in liters; GOLD 0 (FEV_1_ ≥ 80% and FEV_1_/FVC ≥ 0.7) | GOLD 1 (FEV_1_ ≥ 80% and FEV_1_/FVC < 0.7) | GOLD 2 (50% ≤ FEV_1_ < 80% and FEV_1_/FVC < 0.7) | GOLD 3 (30% ≤ FEV_1_ < 50% and FEV_1_/FVC < 0.7) | GOLD 4 (FEV_1_ < 30% and FEV_1_/FVC < 0.7) | PRISm (Preserved Ratio, Impaired Spirometry) (FEV_1_/FVC ≥ 0.7 but FEV_1_ < 80%); history of diabetes, stroke, heart attack, and coronary artery disease based on subject self-report; chronic bronchitis defined by answers to questions about both cough and phlegm. A former smoker was defined by the study visit definition of no smoking in the past 30 days. ANOVA and chi-squared tests were used to examine differences between groups.

**Table 2:** Accelerated age (age_metabolomic_ − age_spline_ > 7) vs. decelerated age (age_metabolomic_ − age_spline_ < −7) in current and former smokers with COPD or emphysema.

	Decelerated (N = 277)	Accelerated (N = 400)	*p*-Value
Chronologic age (years), mean (SD)	67.9 (8.32)	65.3 (8.56)	<0.001
Metabolomic age (years)	58.3 (5.79)	75.2 (6.18)	NA
Race: White/Black/other, %	45.1/53.4/1.5	88.3/10.5/1.4	<0.001
Gender, male, n (%)	197 (71.1%)	172 (43.0%)	<0.001
Smoking Status: Former/current, %	57.8/42.2	75.0/25.0	<0.001
Exacerbations	0 (0, 2.00)	0 (0, 2.00)	0.0178
GOLD stage, n (%)			
GOLD 0	38 (13.7%)	27 (6.8%)	<0.001
GOLD 1	65 (23.5%)	45 (11.3%)	
GOLD 2	90 (32.5%)	131 (32.8%)	
GOLD 3	41 (14.8%)	93 (23.3%)	
GOLD 4	10 (3.6%)	50 (12.5%)	
PRISm	33 (11.9%)	54 (13.5%)	
FEV_1_ (liters)	2.00 (0.801)	1.60 (0.724)	<0.001
Emphysema, %	7.16 (9.45)	10.8 (12.4)	<0.001
FVC	3.21 (0.967)	2.87 (0.938)	<0.001
FEV_1_/FVC	0.613 (0.136)	0.554 (0.160)	<0.001
History of diabetes, n (%)	43 (15.5%)	96 (24.0%)	0.00879
History of stroke, n (%)	12 (4.3%)	29 (7.3%)	0.157
History of heart attack, n (%)	7 (2.5%)	59 (14.8%)	<0.001
History of coronary artery disease, n (%)	11 (4.0%)	79 (19.8%)	<0.001
Chronic bronchitis, n (%)	52 (18.8%)	87 (21.8%)	0.396

Exacerbations included those treated with antibiotics and/or corticosteroids in the 12 months prior to the visit; shown are n (percentages), mean (standard deviations), or medians (5th, 95th percentiles); spirometry volumes are in post-bronchodilator therapy and in liters; GOLD 0 (FEV_1_ ≥ 80% and FEV_1_/FVC ≥ 0.7) | GOLD 1 (FEV_1_ ≥ 80% and FEV_1_/FVC < 0.7) | GOLD 2 (50% ≤ FEV_1_ < 80% and FEV_1_/FVC < 0.7) | GOLD 3 (30% ≤ FEV_1_ < 50% and FEV_1_/FVC < 0.7) | GOLD 4 (FEV_1_ < 30% and FEV_1_/FVC < 0.7) | PRISm (Preserved Ratio, Impaired Spirometry) (FEV_1_/FVC ≥ 0.7 but FEV_1_ < 80%); history of diabetes, stroke, heart attack, and coronary artery disease based on subject self-report; chronic bronchitis defined by answers to questions about both cough and phlegm. *t*-tests and chi-squared tests were used to assess significant differences between the decelerated and accelerated aging groups.

## Data Availability

Clinical and metabolomic data are available through dgGaP (TOPMed) for both COPDGene (phs000179) and SPIROMICS (phs001119).

## Supplementary Materials

The following supporting information can be downloaded at: https://www.mdpi.com/article/10.3390/metabo14120647/s1. Table S1A: Elastic net coefficients for age and lung obstruction models, Table S1B: Aging metabolite sub-pathway enrichment analysis, Table S1C: Aging metabolite super-pathway enrichment analysis, Table S1D: FEV1/FVC metabolite sub-pathway enrichment analysis, Table S1E: FEV1/FVC metabolite super-pathway enrichment analysis, Table S1F: Univariate metabolite associations with age and FEV1/FVC, Table S1G: Sub-pathway analysis of metabolites with significant and concordant univariate associations with age and FEV1/FVC in healthy never/former smokers, Table S1H: Super-pathway analysis of metabolites with significant and concordant univariate associations with age and FEV1/FVC in healthy never/former smokers, Table S1I: Sub-pathway analysis of metabolites with significant and discordant univariate associations with age and FEV1/FVC in healthy never/former smokers, Table S1J: Super-pathway analysis of metabolites with significant and discordant univariate associations with age and FEV1/FVC in healthy never/former smokers, Table S2: Accelerated age (agemetabolomic − agespline > 7) vs. decelerated age (agemetabolomic − agespline < −7) in current smokers without COPD or emphysema, Figure S1: Age acceleration in current smokers without COPD or emphysema.
