# Supplementary material for: Metabolic Aging as an Increased Risk for Chronic Obstructive Pulmonary Disease
Source: Metabolites. 2024 Nov 21;14(12):647. doi: 10.3390/metabo14120647 (PMC11677693; doi:10.3390/metabo14120647)
Supplement: Supplementary file 1 [file metabolites-14-00647-s001.zip › metabolites-3196443-Supplementary_TableS2_FigureS1.pdf]

Supplementary Table S2. Accelerated age ($age_{metabolomic} - age_{spline} > 7$) vs. decelerated age ($age_{metabolomic} - age_{spline} < -7$) in current smokers without COPD or emphysema.

	**Decelerated (N=118)**	**Accelerated (N=31)**	***p*-Value**
Chronologic age (years), mean (SD)	58.6 (6.43)	56.1 (6.26)	0.0536
Metabolomic age (years)	51.9 (4.33)	68.9 (4.52)	NA
Race: White/Black/other, %	20.3/79.7/0	74.2/25.8/0	< 0.001
Gender, male, n (%)	84 (71.2%)	8 (25.8%)	< 0.001
Exacerbations	0 (0, 1.00)	0 (0, 1.00)	0.445
GOLD stage 0, n (%)	118 (100%)	31 (100%)	NA
FEV1 (liters)	2.92 (0.755)	2.67 (0.593)	0.0542
Emphysema, %	0.883 (1.00)	0.885 (1.01)	0.995
FVC	3.73 (0.967)	3.44 (0.843)	0.105
FEV1/FVC	0.786 (0.0491)	0.784 (0.0517)	0.855
History of diabetes, n (%)	13 (11.0%)	8 (25.8%)	0.0694
History of stroke, n (%)	0 (0%)	3 (9.7%)	0.00703
History of heart attack, n (%)	4 (3.4%)	2 (6.5%)	0.796
History of coronary artery disease, n (%)	3 (2.5%)	2 (6.5%)	0.606
Chronic bronchitis, n (%)	20 (16.9%)	12 (38.7%)	0.0128

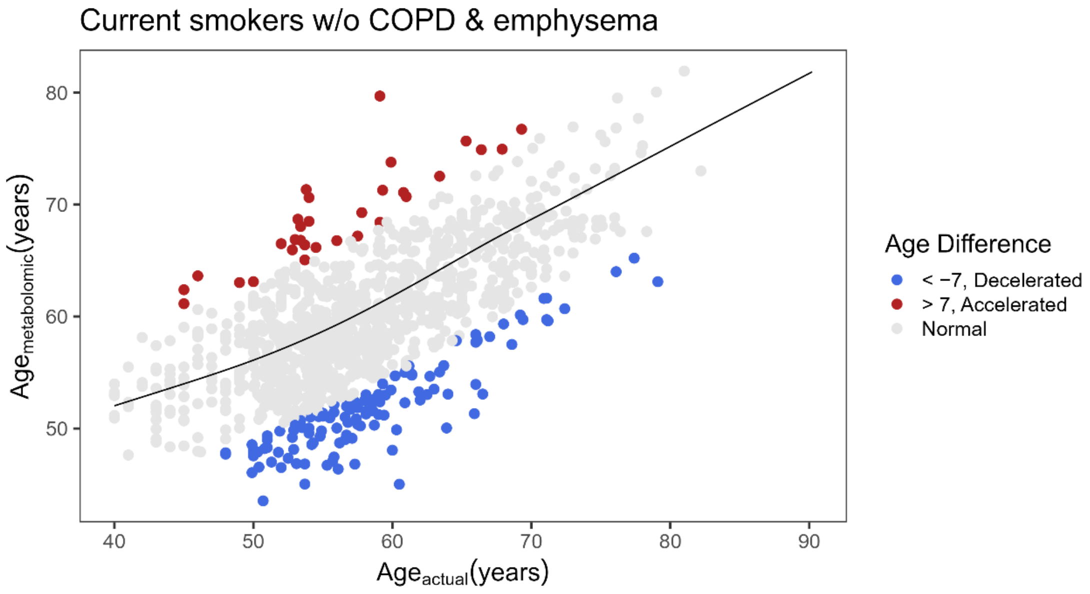

Supplementary Figure S1. Age acceleration in current smokers without COPD or emphysema. A spline was fitted between age$_{actual}$ and age$_{metabolomic}$ (black line). Age difference was calculated by age$_{metabolomic}$ − age$_{spline}$, where subjects having accelerated metabolomic age (red) were those with a difference greater than 7 years and those having decelerated age (blue) were those with a difference less than −7 years.
